# NURSING HOME LEADER PERSPECTIVES OF DELIVERING QUALITY CARE AT TRANSITION TO ENDEMIC PHASE OF COVID-19

**DOI:** 10.1093/geroni/igae098.0082

**Published:** 2024-12-31

**Authors:** LIza Behrens, Kimberly VanHaitsma, Marie Boltz, William Calo, Lauren Van Scov, Jennifer Kraschnewski

**Affiliations:** The Pennsylvania State University, University Park, Pennsylvania, United States; The Pennsylvania State University, University Park, Pennsylvania, United States; The Pennsylvania State University, University Park, Pennsylvania, United States; Penn State College of Medicine, Hershey, Pennsylvania, United States; The Pennsylvania State University College of Medicine, Hershey, Pennsylvania, United States; The Pennsylvania State University College of Medicine, Hershey, Pennsylvania, United States

## Abstract

Nursing Home (NH) leaders are challenged to deliver quality person-centered care (PCC), despite the COVID-19 transition to an endemic phase. This study aimed to describe NH leadership perspectives on preparing and maintaining quality care during the transition point between the COVID-19 pandemic and endemic to identify how best to support NHs in balancing infection risks with PCC. Fifteen (n=15) NH leaders enrolled in the Project Extension for Community Health Outcomes (ECHO) study participated in semi-structured interviews between March and April of 2022. Interviews lasted approximately 60 minutes, were recorded, transcribed verbatim, and verified for accuracy. Multiple coders used constant comparative method to code remaining transcripts (final kappa was.76). Final descriptive themes and subthemes were created via group consensus. NH Leaders reported an average of 18.2 years of experience and held various roles as leaders including Administrator (n=7), Director of Clinical/Health Services (n=3), Director of Nursing (n=2), Director of Infection Prevention and Control (n=1), and Staff Development Director/Coordinator (n=2). Leaders described their perspectives in six themes: Facilitators to operations during COVID-19, Barriers to operations during COVID-19, Resources, Infection control and vaccine uptake, Handling residents’ preferences during COVID-19, and ECHO participation. Results confirmed continued challenges in delivering quality PCC impacted by enforcement issues with vaccination processes and uptake for staff, supply gaps, and lack of knowledge in how to handle residents’ preferences for social activities. Results demonstrate the need for more resources to support staff delivery of quality PCC while balancing risks related to managing the COVID-19 endemic.

